# Cluster Headache in Kuwait: A Hospital-Based Study

**DOI:** 10.3389/fneur.2019.00573

**Published:** 2019-06-04

**Authors:** Jasem Al-Hashel, Ismail Ibrahim, Doaa Youssry, Samar Farouk Ahmed, Peter Goadsby

**Affiliations:** ^1^Department of Neurology, Ibn Sina Hospital, Kuwait City, Kuwait; ^2^Department of Medicine, Faculty of Medicine, Health Sciences Centre, Kuwait University, Kuwait City, Kuwait; ^3^Department of Neurology, Cairo University, Cairo, Egypt; ^4^Department of Neuropsychiatry, Minia University, Minia, Egypt; ^5^Department of Basic and Clinical Neuroscience, Headache Group, Institute of Psychiatry, Psychology and Neuroscience, King's College London, NIHR-Wellcome Trust King's Clinical Research Facility, King's College Hospital, London, United Kingdom

**Keywords:** cluster headache, demographics, trigeminal autonomic cephalalgias, hospital study, Kuwait

## Abstract

**Background:** Cluster headache (CH) is a relatively uncommon primary headache disorder. Few studies in our region described CH. We aimed to study demographics, clinical characteristics and treatment modalities of CH patients referred to Headache Clinic in Kuwait.

**Materials and Methods:** This cross-sectional study included all CH patients who are referred to headache clinic. The diagnosis of CH based on the CH diagnostic criteria of the Headache Classification Committee of the IHS, 3rd edition (beta version) (ICHD-3-beta). The demographics, clinical characteristics and treatment modalities of CH patients were recorded.

**Results:** Forty six patients were diagnosed with CH which constituted 1.7% of all headache patients and 0.1% of all visits to Neurology clinic. Male:Female ratio was 15.3:1. Mean age was 40.1 ± 10.7 years and mean age at onset was 29 years (20–49). Family history was positive in 6.5%. Smoking was seen in 63.0% of patients while 6.5 % reported alcohol intake. CH was episodic in 84.4% and chronic in 15.2%. Seasonal predilection was seen in all patients; the most frequent season was Autumn 47.8%. The mean duration of the cluster bouts was 6 weeks (2–12 weeks). The mean duration of the attack was 60 min (15–180 min). Number of attacks per day ranged from 2 to 10 attacks with a mean of 5 attacks/day. The median of attack severity by Visual Analog Scale (VAS) was 8.5 (7–10). The time taken to diagnose the patients ranged from <1 year to 12 years with a mean of 4 years. Chronic CH have a statically significant relation with smoking (*P* = 0.036), older age (*P* = 0.027), and longer time taken to diagnosis (*P* = 0.026) compared to episodic CH.

**Conclusion:** Our results reported that there is higher proportion of males compared to females and less positive family history. Smoking was a significant risk factor for chronicity in addition to advanced age, higher age at disease onset and a longer time taken till diagnosis.

## Introduction

Cluster headache (CH) is a relatively uncommon yet incapacitating primary headache disorder classified together with other similar conditions as Trigeminal Autonomic Cephalalgias (TAC) (1). The distinguishing features of CH include male predominance, periodic occurrence of cluster attacks (circannual and circadian periodicity), and concurrence of autonomic features (2, 3). The majority of the patients also describe sense of agitation and restlessness during the peak of the headache attack (1).

The attacks of headache tend to cluster in bouts, lasting between 15 and 180 min, recurring from every other day to multiple times per day for weeks or months during active cluster bouts, followed by periods of remission (1, 4). In episodic CH, the bouts often occur with a seasonal pattern separated by headache-free intervals, while in chronic CH the attacks recur for more than a year with no remission or with remission for <3 months (1, 5, 6).

The pain associated with CH can be excruciating and has been described as the most severe pain known to humans (7, 8). Accordingly, the disease has substantial negative impact on social function and quality of life (9).

Few studies in our region described the prevalence and the clinical characteristics of CH and no studies were published from the Arabian Gulf region including Kuwait (10, 11).

We aimed to study demographics, clinical characteristics and treatment modalities of CH patients referred to our Headache Clinic at the only tertiary neurology center in Kuwait during the period from January 2016 to September 2018.

## Materials and Methods

This cross-sectional study included all CH patients who are referred to our headache clinic at the only tertiary neurology center in Kuwait. We reviewed the patient's data registry to identify those with the diagnosis of CH from January 2016, till September, 2018. The patients were contacted and scheduled for reassessment in our headache clinic. The diagnosis of CH was re-challenged and confirmed by headache specialist. All the patients that met the CH diagnostic criteria of the Headache Classification Committee of the IHS, 3rd edition (beta version) (ICHD-3-beta) (1) were included.

Each patient underwent a detailed clinical evaluation and neurological examination by a neurologist. Appropriate brain imaging was requested to rule out secondary headache causes. Demographic data included age, sex, nationality, family history of CH, and history of smoking or alcohol abuse. CH information including type of CH, attack characteristics (site, side, season, severity, character, duration of attack, duration of disease, duration of bouts, frequency), age at the onset, age at diagnosis, associated cranial autonomic features, other associated features (nausea, vomiting, photophobia, phonophobia, aural fullness, and behavioral changes) during attacks and treatment modalities were all recorded. The severity of headache was estimated using a Visual Analog Scale (VAS) to indicate the maximum intensity of pain by asking the patients to make a mark on a 10-cm line labeled with “no pain” at the zero end and “the worst pain possible” at the other (12, 13).

This study was approved by ethical committee of Kuwait ministry of health. Written informed consent was obtained from all participant before using their information.

### Statistical Analysis

All measurements were reported as mean ± SD. Categorical variables were compared using the Chi-square test, and continuous variables were compared using Student's *t*-test. SPSS for Windows, Version 16.0, was used for statistical analyses with the significance level set at *P* = 0.05.

## Results

**Figure 1:**
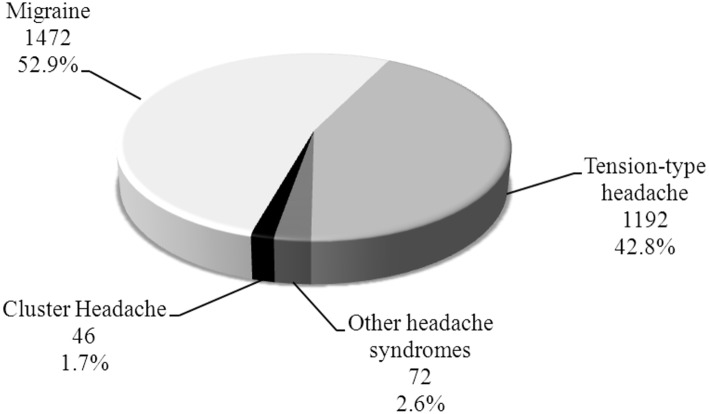
Distribution of headache cases in outpatient population (*n* = 2,782).

A total of 2,782 headache patients were evaluated at our Headache Clinic during the period between January 2016 till September 2018. Forty-six patients were diagnosed with CH which constitute 1.7% of all headache patients (Figure 1) and 0.1% of all patients [45,986] who are reviewed at our Neurology clinics in that period.

### Demographic Characteristics

A total of 46 patients were included, 43 [94.5%] were males and 3 [6.5%] were females, giving a M:F ratio of 15.3:1. Mean age of the patients was 40.1 ± 10.7 years (range 23–69 years). Mean age at disease onset was 29 years (range 20–49 years). Kuwaiti nationality 32 [69.6%] was the most prevalent in our cohort. Twenty-nine [63%] patients were smokers, while only 3 patients reported alcohol intake 6.5%. Family history of CH in a first or second degree relative was reported in only 3 [6.5%] patients (Table 1).

**Table 1:** Demographic profile (*n* = 46).

	**M ± SD/No. (%)**
**Age (years)**	
Mean ± SD	40.1 ± 10.7
**Age at the onset (years)**	
Mean (Range)	29 (20–49)
**Gender**	
Male	43 (94.5)
Female	3 (6.5)
**Nationality**	
Kuwait	32 (69.6)
Egypt	9 (19.6)
Saudi	3 (6.5)
Syria	1 (2.2)
India	1 (2.2)
**Smoking**	29 (63)
**Alcohol intake**	3 (6.5)
**Family history**	3 (6.5)

### Headache Characteristics

**Figure 2:**
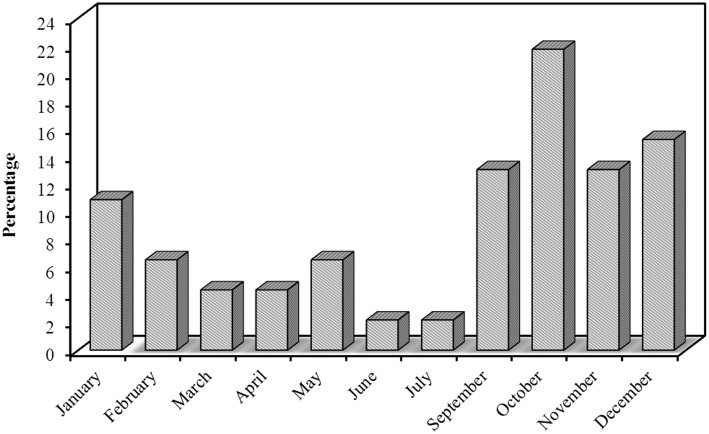
Distribution of the studied cases according to month (*n* = 46).

Most of our patients had episodic CH 39 [84.8%], while only 7 [15.2%] had chronic CH, interestingly all the chronic patients were smokers. CH was strictly unilateral in all our patients with equal right and left side affection. Pain was predominantly affecting peri-orbital and temporal region 43 [93.5%], frontal 2 [4.3%], and occipital 1 [2.2%]. Pain intensity was described by the majority as being severe in 41 [89.1%] patients and moderately-severe in 5 [10.9%] patients. VAS for pain severity was used in all our patients, the score ranged from 7 to 10 with a median of 8.5. Character of pain was throbbing in 35 [76.1%] patients, followed with stabbing 6 [13%], burning 4 (8.7%), and boring in only one 2.2% patient. The duration of the cluster bouts ranged from 2 to 12 weeks with a mean of 6 weeks. The duration of the attack ranged from 15 to 180 min with a mean of 60 min. The number of attacks per day ranged from 2 to 10 attacks with a mean of 5 attacks/day. Regarding the frequency of cluster periods, the majority had one bout per year; 25 [54.3%] patients, every 2 years in 10 [21.7%], every 3 years in 2 [4.3%], every 3 months in 4 [8.7%], every month in 4 [8.7%], and every 6 months in only one (2.2%) patient. Seasonal predilection was observed in our patients; the most frequent season was Autumn 22 [47.8%] patients, followed with Winter 15 [32.6%], Spring 7 [15.2%], and the least frequent was Summer 2 [4.3%] patients. These cluster periods occurred mainly in October, December, September, and November (Figure 2).

The time taken to diagnose ranged from <1 to 12 years with a median of 4 years. The total duration of the disease among our patient ranged from 2 to 30 years with a mean of 10 years. Among cranial autonomic features, rhinorrhea and nasal congestion were found in all 46 [100%] patients, tearing was found in 45 [97.8%] patients, conjunctival injection in 44 [95.7%], ptosis and miosis were found in 10 (21.7%), while facial sweating in only 4 [8.7%] patients. Other associated features during the clusters were nausea with or without vomiting in 28 (60.9%) patients, photophobia and phonophobia in 23 [50%] patients, of which 20 [43.5%] patients reported it as unilateral. Agitation during the attacks was reported by 41 [89.1%] patients. Aural fullness was reported by 28 [60.9%] patients.

Neurological examination between the cluster attacks was normal in all patients 100%. All patients underwent magnetic resonance (MR) imaging of the brain; 41 [89.1%] patients were normal, non-specific white matter changes (WM) were reported in 4 (8.7%) patients, and one patient 2.2% had incidental sellar cyst.

In our cohort, some patients received the diagnosis of another type of headache in addition to CH, 8 [17.4%] patients had migraine, 4 [8.7%] patients had tension-type headache and one patient 2.2% had mixed type of migraine and tension headache. Two patients were diagnosed with secondary headache; one patient had a sellar cyst and another one had foreign bodies in the skull and left maxillary sinus during the Gulf War (Table 2).

**Table 2:** Characters of headache pain (*n* = 46).

	**No. (%)/Mean (Range)**
**Type of CH**	
Episodic	39 (84.8)
Chronic	7 (15.2)
**Side**	
Right	23 (50)
Left	23 (50)
**Site**	
Periorbital-temporal	43 (93.5)
Frontal	2 (4.3)
Occipital	1 (2.2)
**Season**	
Autumn	22 (47.8)
Winter	15 (32.6)
Spring	7 (15.2)
Summer	2 (4.3)
**Severity**	
Moderate-severe	5 (10.9)
Severe	41 (89.1)
**VAS Severity Scale**	
Median (range)	8.5 (7–10)
7	3 (6.5)
8	20 (43.5)
9	19 (41.3)
10	4 (8.7)
**Character**	
Throbbing	35 (76.1)
Stabbing	6 (13)
Burning	4 (8.7)
Boring	1 (2.2)
**Duration of attack (min)**	60 (15–180)
**Duration of the disease (years)**	10 (2–30)
**Duration of cluster bout (weeks)**	6 (2–12)
**Frequency**	
Once/1 month	4 (8.7)
Once/3 months	4 (8.7)
Once/6 months	1 (2.2)
Once/1 year	25 (54.3)
Once/2 years	10 (21.7)
Once/3 years	2 (4.3)
**Age of disease onset (years)**	29 (20–49)
**Time taken till diagnosis (years)**	4 (0–12)
**Associated symptoms**	
Rhinorrhea	46 (100)
Nasal congestion	46 (100)
Lacrimation	45 (97.8)
Aural fullness	22 (47.8)
Conjunctival injection	44 (95.7)
Facial sweating	4 (8.7)
Ptosis	10 (21.7)
Miosis	10 (21.7)
Agitation	41 (89.1)
Nausea/Vomiting	28 (60.9)
Photophobia & phonophobia	23 (50)
**Associated headaches**	
No	33 (71.7)
Migraine	8 (17.4)
TTH	4 (8.7)
**Examination/Normal**	46 (100)
**MRI/MRA**	
Normal	41 (89.1)
Non-specific WM changes	4 (8.7)
Seller cyst	1 (2.2)

*CH, Cluster Headache; VAS, Visual Analog Scale; TTH, tension type headache; MRI, Magnetic resonance image; MRA, Magnetic resonance angiography; WM, white matter*.

### Chronic CH

We studied the relationship between different headache characteristics and chronicity in CH. Age of chronic CH patients (48.3 ± 11.3) was higher than episodic CH patients (38.7 ± 10.1), the difference was statistically significant (*P* = 0.027).

Age at disease onset was also higher in chronic CH patients; 31years (24–46) compared to episodic CH patients; 27 years (20–49) with a statistically significant difference (*P* = 0.036). Smoking was reported by 100% of chronic cases compared to 56.4% of episodic cases which was statistically significant (*P* = 0.036). The mean time taken till diagnosis was 4 years in episodic CH and 6 years in chronic CH which was statistically significant (*P* = 0.026) (Table 3).

**Table 3:** Association between type of CH and headache characteristics.

	**Type of CH**		***p***
	**Episodic (*n* = 39)**	**Chronic (*n* = 7)**	
Age	38.9 ± 9.8	48.1 ± 11.3	0.029^*^
Smoking	22 (56.4%)	7 (100.0%)	0.036^*^
Time taken till diagnosis	4 (0–10)	6 (3–12)	0.026^*^
Age of onset of the disease	27 (20–49)	31 (24–46)	0.036^*^

* *P < 0.05 is significant*.

### Treatment

Treatment was individualized for each patient. During acute attacks, all our patients received high-flow oxygen. Steroids either Dexamethasone or Prednisone were given to 45 [97.8%] patients. Triptans were given to 37 [80.4%] patients; sumatriptan being the most commonly used by 32 (69.6%) patients. Forty patients 87% reported using non-steroidal anti-inflammatory drugs (NSAIDs) during acute attacks. For preventive treatment, the most common drug used was topiramate in 24 [52.2%] patients followed by verapamil in 22 [47.8%] and lithium in one patient 2.2%. Other drugs used included Tricyclic antidepressants (TCA) in 4 [8.7%] patients and valproic acid in 1 [2.2%]. Greater occipital nerve block (GONB) and Botulinum toxin (BTX) were given to 6 [13.0%] patients each. Only one patient 2.2% in our cohort underwent Percutaneous radiofrequency ablation (RFA) of the sphenopalatine ganglion (SPG) due to poor response to medical treatment (Table 4).

**Table 4:** Treatment modalities (*n* = 46).

	**No. (%)**
**ACUTE TREATMENT**
High flow oxygen	46 (100)
Steroids	45 (97.8)
Triptans	37 (80.4)
NSAID	40 (87)
**PROPHYLACTIC TREATMENT**
Topiramate	24 (52.2)
Verapamil	22 (47.8)
TCA	4 (8.7)
Lithium	1 (2.2)
Valproic acid	1 (2.2)
**OTHERS**
GONB	6 (13)
BTX	6 (13)
SPG RFA	1 (2.2)

*NSAID, Non-steroidal anti-inflammatory drugs; TACs, Trigeminal Autonomic Cephalalgias; GONB, Greater occipital nerve block; BTX, botulinum toxin; RFA, Radiofrequency ablation; SPG, Sphenopalatine ganglio; nTCA, Tricyclic antidepressants; RFA, Radiofrequency ablation*.

## Discussion

CH data from our region in general are scarce in literature and no data were previously published from gulf countries (10, 11).

Prevalence of CH in our cohort was 1.7% of all headache patients evaluated in our clinic which matches with the prevalence found in other clinic-based studies from Egypt 1.7% (14), China 1.7% (15), Pakistan 1.6% (16), and Ethiopia 1.3% (17). Accurate estimation of CH prevalence is difficult because of its low prevalence in general population in comparison to migraine and tension-headache (18). CH constituted 0.1% of all patients evaluated in our neurology outpatient clinics during the duration of the study, while in china the frequency of CH in the neurological outpatient clinic population was 0.2% (15). The prevalence of CH in community-based studies ranged from 0.1 to 0.2% (19, 20).

CH attacks affect patients in their most productive years with negative results on their quality of life (9, 15). The mean age of our patients was 40.1(±10.7) years and the mean age at onset was 29 (± SD 5.3) years. Our results are in consistence with previous studies (15, 21, 22) the mean age of onset in China (27 ± 8) (15), in Taiwan (26.9) years (21), in Japan (31) years (22), and in USA 36% of patients were 20–30 years old (23).

CH was more common in males than in females in our cohort with male-to-female ratio of 15.3:1 which is higher than any figure published before. The male-to-female ratio is variable in literature being generally higher in Asian studies and lower in Western studies ranging from 14:1 to 2.5:1 (15, 21, 22). In patient sample studies, this ratio ranged between ranged between 2.5:1 and 14:1 (20, 21, 24–27). In the Vågå study of headache epidemiology including CH, female to male was one to six (28).

The high male to female ratio in our cohort may be related to the conservative nature of our population with low smoking prevalence among women and alcohol being prohibited. Another possible explanation can be related to the climate and latitude effect on CH which is not well studied so far.

Smoking is considered a significant risk factor for developing and triggering CH (21, 23). In our cohort, more than 60% were smokers. The majority of US CH patients had a history of smoking (73% total patients, with 51% actively smoking at time of CH onset) (18). Schürks and Diener reported that 94% of the patients were smokers, with an average of 32 cigarettes smoked per day (29). Tiraferri et al. found 90% of CH patients had a prolonged history of smoking (30). All chronic CH patients in our cohort were smokers. Current smoking history was significantly more prevalent among chronic CH patients more than episodic CH patients [75 vs. 65%] in a British study (31) and 70.8 vs. 65.2% in a German study (26). These findings point to not only the association between smoking and CH, but to a possible causal association that deserves mentioning as well (26), moreover, improvement after reducing their cigarette consumption strongly support such hypothesis (29). Alcohol intake as another known trigger (29) was reported by only 6.5% of our patients as its consumption is prohibited in Kuwait.

Prevalence of CH in the same family is variable in different studies. We reported 6.5% of our patients have history of headache in at least one of their family members. Positive history reported in 1.9–6.7% in Bharar et al patients (24), other studies showing that 7% of CH patients had a positive family history of headache (31), while Rozen and Fishman reported that around 18% of CH patients in US had positive family history (23). CH risk in first-degree relatives is 5–18 times higher, and in second degree relatives, 1–3 times higher as compared to the general population (18, 30).

The majority of our cases had episodic CH 84.4% which is in agreement with Japanese studies that reported a low prevalence of chronic CH (1.2–2.9%) (22), while other studies from Asia reported no chronic cases at all (15, 21). Large-scale population-based studies are necessary to determine if chronic CH is truly less prevalent in Kuwait as seen in other Asian populations.

All of the patients had a strictly unilateral headache with no side predominance, Xie et al. and Wang et al. reported right-sided predominance (15, 21), other studies reported side shift in some of their patients (23, 26).

Seasonal predilection is common in CH which is considered a circadian-based syndrome (18). In our study, the majority of our patients presented in autumn and winter with the highest prevalence in October and December, the least presentation was in the summer. This pattern was first noted by Ekbom and Kudrow and was repeatedly seen over the years (18, 32, 33).

It is well known that CH is one of the worst headaches and this was agreed upon by 89.1% of our patients who reported having severe rather than moderately severe headache with a mean VAS of 8.5. These results were similar to another clinic-based study from China where the mean VAS was 8.2 (15).

The associated autonomic symptoms which are the defining feature of CH were found in all of our patients similar to other studies. Rhinorrhea 100% and nasal congestion 100% were the most reported symptoms in all of our patients followed by conjunctival injection and lacrimation while ptosis and miosis were the least reported. Lacrimation was the most reported associated autonomic symptom in some studied (13, 34), and less frequens in other studies (15). A sense of restlessness or agitation, which differentiate CH from migraine was seen in 89.1% of our patients which is in agreement with previous studies that reported it ranged between 67.6 and 93% (22, 24, 26).

Photophobia and/or phonophobia were reported in half of our patients and interestingly most of them reported it being unilateral at the side of pain which was previously reported by Irimia et al. (35).

The mean time taken till accurate diagnosis in our cohort was 4 years. In a Dutch study the median time before a proper diagnosis of CH was 3 years (36), Klapper et al. found a time delay of 6.6 years in a US population (37), Rozen et al. study showed a 5 years delay or longer in around half of the patients (28). Factors that may lead to delay are the presence of photophobia or phonophobia, nausea or vomiting which may lead to misdiagnosis as migraine, an episodic attack pattern and a younger age at onset (36). In hospital bases study of Cristina Voiticovschi-Iosob showed that the mean interval between onset of the disease and first consultation at a headache center was 4.1 ± 5.6 years. Their patients had consulted different specialists prior to receiving their CH diagnosis: neurologists (49%), primary care physicians (35%), ENT specialists (10%), dentists (3%). Misdiagnoses at first consultation were recorded in 77% of the cases: trigeminal neuralgia (22%), migraine without aura (19%), sinusitis (15%). The average “diagnostic delay” was 5.3 ± 6.4 years (38). Nine (19.5%) patients in our cohort were initially misdiagnosed as migraine that changed to CH over the years.

Treatment of CH in our cohort followed an individualized approach. During acute attacks, all our patients received high flow oxygen with good response. The majority also received steroids 79.8% either oral or parenteral during the attacks and reported using over the counter non-steroidal anti-inflammatory drugs. Sumatriptan was the commonest triptan to be used as it has both oral and subcutaneous route of administration. Topiramate was the commonest prophylactic treatment used followed by verapamil. BTX injection and GONB were both given to some of our patients who showed inadequate response to medical treatment and were found to be a safe and effective option in several studies with good outcome (39–41). RFA-SPG was performed for only one patient with secondary CH who failed medical treatment and he showed favorable outcome (3).

## Limitations of the Study

Our study had some limitations being a hospital-based study including: the small sample size and the lack of impact of CH on our patient's occupation and quality of life. Our cohort is small because CH is uncommon primary headache disorder in addition that it might be misdiagnosed as sinus headache by general practioner before referral to our specialized headache clinic. Health service in Kuwait start in primary care, then refractory cases are referred to secondary and tertiary services.

## Strength of Our Study

This study has the strength of providing data from Kuwait where the prevalence of CH could be different from that in the general population, perhaps as a consequence of traditions and religion (e.g., smoke and alcohol).

## Conclusion

This study describes the demographic and clinical characteristics of CH in a neurology outpatient in Kuwait. Our results coincided with other clinical studies published in many aspects, including the prevalence and clinical characteristics of CH. However, it was different in the highest proportion of men and in a less positive family history. We found that smoking is a significant risk factor for chronicity in patients with CH in addition to advanced age, higher age at disease onset and a longer time taken till diagnosis. Further community-based studies are needed in the gulf region to give better understanding of CH in this part of the world.

## Data Availability

Data are available at neurology department, Ibn Sina hospital, Kuwait.

## Ethics Statement

Our research was carried out according to ethical guidelines of Kuwait Ministry of Health.

## Consent for publication

All authors have read and approved the submitted manuscript. Our manuscript has not been submitted elsewhere nor published elsewhere.

## Author Contributions

JA-H designed the study and reviewed the manuscript. II performed data collection, statistical analysis and drafted the manuscript. DY performed data collection and drafted the manuscript. SA drafted, criticized and reviewed the manuscript. PG reviewed the manuscript. All authors read and approved the final manuscript.

### Conflict of Interest Statement

The authors declare that the research was conducted in the absence of any commercial or financial relationships that could be construed as a potential conflict of interest.

## Abbreviations

BTX: Botulinum toxin
CH: Cluster Headache
GONB: Greater occipital nerve block
ICHD: International Classification of Headache Disorders
MR: Magnetic resonance
NSAID: Non-steroidal anti-inflammatory drugs
RFA: Radiofrequency ablation
SPG: Sphenopalatine ganglion
TACs: Trigeminal Autonomic Cephalalgias
TCA: Tricyclic antidepressants
VAS: Visual Analog Scale.
